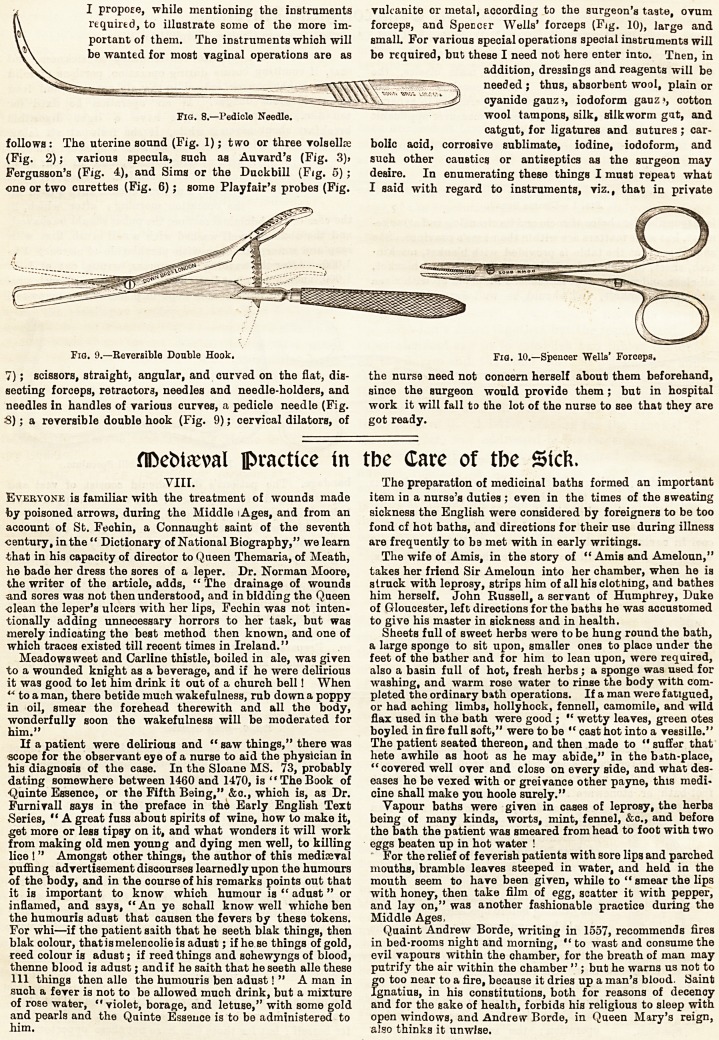# "The Hospital" Nursing Mirror

**Published:** 1898-10-01

**Authors:** 


### "The Hospital" Nursing Mirror.

Being the Nursing Section of "The Hospital."

[Contributions for this Section of "The Hospital" should be addressed to the Editor, The Hospital, 28 & 29, Southampton Street, Strand, London, W.C., and should have the word "Nursing" plainly written in left-hand top corner of the envelope.]

### News from the Nursing World.

### THE QUEEN REGENT OF SPAIN AND HER SOLDIERS.

The Queen Regent of Spain has all through the war shown the most intense sympathy with the sick and wounded of the Spanish army. She visits daily those who are in hospital at Madrid, and those who are convalescent are conveyed to the Casa del Casa Campo in the Royal Park in the Queen's carriages. Daily supplies of milk are sent to the sick soldiers from the Royal dairies, and in every possible way Her Majesty strives to lessen their sufferings by tokens of womanly kindness. The Princess Mercedes, too, would not this year celebrate her birthday by a State ball, but requested that the anniversary might be marked by her assumption of the office of President of the Spanish Red Cross Society. The Princess at the same time gave a dinner to the wounded soldiers who had returned from the front.

### CONFERENCE OF WOMEN WORKERS.

The annual conference of the National Union of Women Workers is to be held on October 25th, 26th, 27th, and 28th, at the Prince's Street Rooms, Norwich. Several of the subjects to come under discussion will be of special interest to nurses. On Tuesday, the 25th, the papers will be on "The Work of Midwives in the Villages," by Mrs. Wallace Bruce and Miss Katharine Twining, and the discussion will be opened by Lady Laura Ridding and Miss Hughes, of the Nurses' Cooperation. On the second day, Miss Gibson, matron of the Birmingham Infirmary, will read a paper on "The Care and Nursing of the Epileptic"; and others will be upon "The Care and Education of Deaf Mutes," and "The Care and Training of the Feeble-minded." Further particulars can be obtained from the organising secretary, Miss Janes, at the office, 59, Berners Street, W.

### NUNS AT THE FRONT.

There has certainly been no lack of nursing material at the disposal of the military authorities on both sides during the late war. How great a change from Crimean times, when many good people were positively shocked at the "impropriety" of women going out to the seat of war to nurse the sick and wounded! The various Roman Catholic Orders of nursing nuns were prompt in their offers of assistance, and the services of nearly a hundred of the good Sisters were accepted at different times. When President McKinley was informed that Mother Marianna, Superior of an Order having its head-quarters at Emmittsburg, was in Washington, he asked her to call at White House that he might personally express to her his sense of obligation for her offers of help. The President told the Reverend Mother, and Sister Beatrice, superintendent of the Providence Hospital, who accompanied her to the White House, that he well remembered the good work done by the Sisters during the Civil War, and he was glad to welcome them back to the army again.

### THE LONDON TEMPERANCE HOSPITAL.

The Board of Management of the London Temperance Hospital have appointed Miss A. D. Lucas to the post of matron in succession to Miss Orme, and the choice is one upon which they are to be congratulated. Miss Lucas has held the post of sister-housekeeper at the Temperance Hospital for several years past, and done excellent work in that capacity. Trained at the Leeds General Infirmary, she was for some years sister of a male ward and of the theatre at the Radcliffe Infirmary, Oxford, besides having had other varied experience. Miss Orme takes her farewell of the hospital where she has worked for so long on Friday, October 7th, when the Board have issued invitations for a social gathering "to join in a recognition" of her "official services" from October 3rd, 1873, to the present time.

### DAILY VISITING NURSING.

Miss Lowdell has begun acting as daily visiting nurse in the neighbourhood of West Kensington. Her terms are most reasonable, and the boon, to the class of patients which she hopes to reach, is undeniable. Every new thing takes time to inaugurate, but once a good nurse is known she must have more work than she can manage. Mothers will be able, with the nurses' periodical visits, to attend to their own children, and daughters will, with a nurse's advice and help, be enabled to nurse any simple ailment, and at the same time acquire a knowledge of home nursing, which is always a valuable accomplishment. Miss Lowdell's address is 9, Avonmore Gardens, West Kensington.

### LECTURES ON "BUSINESS" AT THE TRAINED NURSES' CLUB.

A most helpful course of lectures is about to be initiated at the Midwives' Institute and Trained Nurses' Club, 12, Buckingham Street, Strand. It was suggested some time ago that classes for "instruction in business details" would prove valuable to many nurses and others, whose training has necessarily lacked much that the busy women of to-day are constantly needing, and the idea met at once with so much encouragement that it is to be put into operation without delay. The classes will probably begin early in October, on Mondays or Tuesdays and Thursdays, at half-past five p.m. The fee for the course of lectures will be 2s. 6d. to members of the club, 5s. to non-members; members can attend single lectures for 6d., non-members for 1s. Application for tickets and the syllabus should be made to the hon. secretary, Miss Toynbee, as soon as possible, and any suggestions from intending pupils will be gladly received and considered. The lecturer is to be Miss

Somerville, of the Women's Institute, who has had a large experience in training women to fill business positions.

### NURSES AND CYCLING.

In our columns we reported the remarks of a correspondent on an advertisement for non-cycling nurses. We have heard from the institution in question that there is no room for bicycles, and also that the matron objects to bicycling for women, and feels that she has a right to prohibit the recreation amongst the nurses of her staff.

### GLOUCESTER DISTRICT NURSING SOCIETY.

The announcement of Miss Evans' resignation of her post as superintendent of the District Nursing Society at Gloucester will have been heard with much regret by all who know how excellent has been her work there for the past eight years. The sick poor of Gloucester will not soon forget all her labours on their behalf during the small-pox epidemic; and Miss Evans will carry with her to Bangor, where she goes this month to take up somewhat lighter duties, very cordial good wishes from many quarters. The committee have secured the services of Miss Emma Dudley as their new superintendent, a lady of much experience in the management of district work. Notably she has been for some time past assistant superintendent at the Metropolitan Nursing Association's Home in Bloomsbury Square.

### THE LITTLE HOPPERS' HOSPITAL.

Last year Father Richard Wilson, of St. Augustine's, Stepney, spent the hopping season amongst his pickers from Stepney in the Kentish gardens, an experience which led him this summer to issue an appeal for help to open there a hospital for the sick and ailing amongst the children. He took a cottage at Five Oak Green, near Paddock Wood, fitted it up with cots and cradles, placed it in charge of two trained nurses, and opened the tiny place as "The Little Hoppers' Hospital." A correspondent of a contemporary tells of the boon this has been, and adds "There are seven beds, and they have been nearly always filled; two women have been received beside the children, both of them at the time in a dying state, and their last moments were soothed and quieted in the wards of the L.H.H." A neighbouring doctor paid daily visits, and much good work was also done amongst the out-patients, who came to have their various ills and maladies attended to.

### TRAINED NURSING IN ADELAIDE.

The fourth annual meeting of the District Trained Nursing Society, Adelaide, was held at the Victoria Hall, Sir T. Fowell Buxton (the Governor of the Colony) presiding. The first thing that strikes one on reading the report is that the cost of the trained nurse is the same there as in England—namely, £80 a year. The remuneration is not sufficient, therefore, to induce English nurses to seek work in that direction, and bears out what is frequently stated, that trained nurses without means are better off at home, unless they have other reasons than money-making for emigrating. We note with pleasure that the work is growing, more nurses are employed, wider districts are covered by the operations of the society, and negotiations are proceeding with relation to the amalgamation of another similar society working in East Adelaide. An appeal has been issued by the committee to the various churches, asking for collections in aid of the funds, and the staff of nurses maintains an excellent standard, both of work and character.

### INDIGNATION AT COOLGARDIE.

Considerable public indignation and excitement has been aroused at Coolgardie in consequence of the dismissal of several nurses from the Government Hospital, and at a largely attended meeting, presided over by the Mayor, a motion was unanimously carried urging a reconsideration of the matter upon the premier. A copy of the resolution passed was ultimately presented to the Colonial Secretary, and investigation into the affair will doubtless follow.

### DIFFERENCE A DISTINCTION.

An amusing grievance occupies the attention of the Governors of the Monoghan and Cavan Lunatic Asylum at the moment. Once upon a time the nurses and attendants dined together, but the resident medical superintendent saw fit to establish two tables, the one for the nurses and the other for the attendants; and this is taken by the staff to be an imputation against its character. The privilege of protest belongs to all, and the nurses and attendants of the asylum availed themselves of it. A letter, praying for the renewal of the old order, was addressed to the Governors, which, however, has failed to produce the desired effect, or to awaken remorse in the stony heart of the medical superintendent, who said, when questioned, that the present arrangement is the better one, and that if the institution had been properly built there would have been separate dining halls instead of separate tables. The letter was marked "read."

### ARMAGH GUARDIANS AND THE LOCAL GOVERNMENT BOARD.

The Armagh Board of Guardians and the Irish Local Government Board are at it tooth and nail. The report of the inspector has revealed a frightful state of things—frightful even for an Irish workhouse. The nursing, or rather the lack of it, in any proper meaning of the word, and the insanitary state of the premises generally, have brought about a strong condemnation from Mr. Agnew, and consequent recommendations and commands from the Local Government Board, which the Guardians are at present defying. Meanwhile, one can only feel sincere sympathy with the unfortunate paupers.

### SHORT ITEMS.

The matron of the Salisbury Infirmary has received from Mr. Seth Chinnock, a farmer, the sum of £5 as a contribution towards an outing for the nurses. For two years past funds have not sufficed to provide the nurses with this annual treat, so that the gift is particularly welcome.—Four district nurses are employed by the Grimsby and District Nursing Institution, and the pressure of work has been so heavy of late that extra help has been required. But it is stated that unless further subscriptions are soon forthcoming it will be impossible to keep up the staff, great as is the need in the district.—On October 3rd two district nurses begin work amongst the sick poor of the parishes of Isleworth and Hounslow, under the auspices of a newly-started Nursing Association. To carry on the work a sum of £200 per annum will be necessary, towards which the Isleworth Charity Board has promised £80, providing the association can find the remainder.

### Notes on Gynæcological Nursing.

By Arthur E. Giles, M.D., B.Sc., F.R.C.S., Assistant Surgeon Chelsea Hospital for Women.

### IV.—PREPARATION FOR OPERATION.

Gynæcological operations are of two main classes, the vaginal and the abdominal. I shall consider first the preparations required for operations in general, and afterwards the special measures applicable to each class of operation.

Fig. 1.—Uterine Sound.

(1) Preparation of the Room.—The surgeon is responsible for choice of room and its cleansing and arrangement, but some matters are within the nurse's province. She should see that the table is provided with blanket, mackintosh, sheet, pillows, and a light coverlet or second blanket, all clean and warm. The bed should be made up with clean sheets and blankets, and should be well warmed by means

Fig. 2.—Volsella.

of hot-water bottles. There should be several of these available also on the table during operation. The nurse will see that there is plenty of boiling water to be had, as well as water that has been boiled and allowed to cool in covered vessels. One or two small tables covered with clean towels will be useful for holding instruments,

Fig. 3.—Auvard's Speculum.

sponges, and dressings; some wide shallow dishes for instruments, bowls for sponges and for lotions, and small dishes or bowls for needles and suture material; plenty of towels; a foot-bath or stoneware slop-pail for catching discharges, irrigation fluid, or the contents of ovarian cysts; all these the nurse should see to. She should also prepare a nutrient enema of brandy and beef-tea, in case it is required for administration before the patient is put back to bed.

Fig. 4.—Fergusson's Speculum.

(2) Preparation of the Patient.—On the evening before operation the patient should have an aperient; whatever she is accustomed to take will usually answer best. On the morning of the operation day an enema should be given. No solid food should be given for some hours before operation; the reason given for this is that it would increase the tendency to sickness, and that, if vomiting occurs during operation, portions of solid matter might become lodged in the air passages and lead to considerable danger. If an operation be fixed for ten a.m., the patient might have a light digestible breakfast about seven; while, if she feels at all faint, she can have a few mouthfuls of beef tea, either plain or with a little brandy, half an hour to an hour before operation. Whenever it is possible, a patient should have a bath on the operation morning; after which, in the case of an abdominal section, the pubes should be shaved, and the abdomen well washed with a nail brush, first with soap and water, and then with perchloride of mercury 1 in 1,000. After the washing a compress, wrung out of 1 in 1,000, should be placed on the abdomen, covered with protective or mackintosh, and kept in place with a many-tail

Fig. 5.—Sims' Duckbill Speculum.

bandage. The patient's dress should consist of vest and nightdress, and either a petticoat which can be slipped down below the hips during operation, or, better still, a pair of long white woollen stockings which will reach to the top of the thighs. This arrangement is specially desirable for vaginal operations, where it may be necessary for the patient to be exposed for some time.

The difference in the preparations for an abdominal and a vaginal operation consists mainly in the difference of the field of operation to be prepared. The preparation of the abdomen in the former kind of case has been just referred to. Preparation of the operation field for a vaginal operation consists mainly of douching. Of this I shall have

Fig. 6.—Sims' Curette.

something more to say in a subsequent lecture. Shaving of the vulva is generally done, when necessary, under the anæsthetic, just before operation is begun. The last thing before the patient begins to take the anæsthetic is that the bladder should be emptied, naturally if possible, but, if necessary, with the catheter. It should be remembered that there are comparatively few cases in which the natural emptying of the bladder will not suffice.

(3) Instruments for a Vaginal Operation.—As a rule, it is only in hospital that a nurse would have to get instruments ready and select those wanted for particular operations; in private this is done by the surgeon himself. Still it is advisable that a nurse should make herself familiar with

Fig. 7.—Playfair's Probe.

the names and uses of the several instruments. This is, of course, best learnt by her seeing them and handling them for herself; but for those who have not these opportunities,

FIG. 8.—Pedicle Needle.

I propose, while mentioning the instruments required, to illustrate some of the more important of them. The instruments which will be wanted for most vaginal operations are as follows: The uterine sound (Fig. 1); two or three volsellæ (Fig. 2); various specula, such as Auvard's (Fig. 3), Fergusson's (Fig. 4), and Sims or the Duckbill (Fig. 5); one or two curettes (Fig. 6); some Playfair's probes (Fig. 7); scissors, straight, angular, and curved on the flat, dissecting forceps, retractors, needles and needle-holders, and needles in handles of various curves, a pedicle needle (Fig. 8); a reversible double hook (Fig. 9); cervical dilators, of vulcanite or metal, according to the surgeon's taste, ovum forceps, and Spencer Wells' forceps (Fig. 10), large and small. For various special operations special instruments will be required, but these I need not here enter into. Then, in addition, dressings and reagents will be needed; thus, absorbent wool, plain or cyanide gauze, iodoform gauze, cotton wool tampons, silk, silkworm gut, and catgut, for ligatures and sutures; carbolic acid, corrosive sublimate, iodine, iodoform, and such other caustics or antiseptics as the surgeon may desire. In enumerating these things I must repeat what I said with regard to instruments, viz., that in private the nurse need not concern herself about them beforehand, since the surgeon would provide them; but in hospital work it will fall to the lot of the nurse to see that they are got ready.

FIG. 9.—Reversible Double Hook.

FIG. 10.—Spencer Wells' Forceps.

### Mediæval Practice in the Care of the Sick.

VIII.

EVERYONE is familiar with the treatment of wounds made by poisoned arrows, during the Middle Ages, and from an account of St. Fechin, a Connaught saint of the seventh century, in the "Dictionary of National Biography," we learn that in his capacity of director to Queen Themaria, of Meath, he bade her dress the sores of a leper. Dr. Norman Moore, the writer of the article, adds, "The drainage of wounds and sores was not then understood, and in bidding the Queen clean the leper's ulcers with her lips, Fechin was not intentionally adding unnecessary horrors to her task, but was merely indicating the best method then known, and one of which traces existed till recent times in Ireland."

Meadowsweet and Carline thistle, boiled in ale, was given to a wounded knight as a beverage, and if he were delirious it was good to let him drink it out of a church bell! When "to a man, there betide much wakefulness, rub down a poppy in oil, smear the forehead therewith and all the body, wonderfully soon the wakefulness will be moderated for him."

If a patient were delirious and "saw things," there was scope for the observant eye of a nurse to aid the physician in his diagnosis of the case. In the Sloane MS. 73, probably dating somewhere between 1460 and 1470, is "The Book of Quinte Essence, or the Fifth Being," &c., which is, as Dr. Furnivall says in the preface in the Early English Text Series, "A great fuss about spirits of wine, how to make it, get more or less tipsy on it, and what wonders it will work from making old men young and dying men well, to killing lice!" Amongst other things, the author of this mediæval puffing advertisement discourses learnedly upon the humours of the body, and in the course of his remarks points out that it is important to know which humour is "adust" or inflamed, and says, "An ye schall know well whiche ben the humouris adust that causen the fevers by these tokens. For whi—if the patient saith that he seeth blak things, then blak colour, that is melencolie is adust; if he se things of gold, reed colour is adust; if reed things and schewyngs of blood, thenne blood is adust; and if he saith that he seeth alle these 111 things then alle the humouris ben adust!" A man in such a fever is not to be allowed much drink, but a mixture of rose water, "violet, borage, and letuse," with some gold and pearls and the Quinte Essence is to be administered to him.

The preparation of medicinal baths formed an important item in a nurse's duties; even in the times of the sweating sickness the English were considered by foreigners to be too fond of hot baths, and directions for their use during illness are frequently to be met with in early writings.

The wife of Amis, in the story of "Amis and Ameloun," takes her friend Sir Ameloun into her chamber, when he is struck with leprosy, strips him of all his clothing, and bathes him herself. John Russell, a servant of Humphrey, Duke of Gloucester, left directions for the baths he was accustomed to give his master in sickness and in health.

Sheets full of sweet herbs were to be hung round the bath, a large sponge to sit upon, smaller ones to place under the feet of the bather and for him to lean upon, were required, also a basin full of hot, fresh herbs; a sponge was used for washing, and warm rose water to rinse the body with completed the ordinary bath operations. If a man were fatigued, or had aching limbs, hollyhock, fennell, camomile, and wild flax used in the bath were good; "wetty leaves, green otes boyled in fire full soft," were to be "cast hot into a vessille." The patient seated thereon, and then made to "suffer that hete awhile as hoot as he may abide," in the bath-place, "covered well over and close on every side, and what deseases he be vexed with or greivance other payne, this medicine shall make you hoole surely."

Vapour baths were given in cases of leprosy, the herbs being of many kinds, worts, mint, fennel, &c., and before the bath the patient was smeared from head to foot with two eggs beaten up in hot water!

For the relief of feverish patients with sore lips and parched mouths, bramble leaves steeped in water, and held in the mouth seem to have been given, while to "smear the lips with honey, then take film of egg, scatter it with pepper, and lay on," was another fashionable practice during the Middle Ages.

Quaint Andrew Borde, writing in 1557, recommends fires in bed-rooms night and morning, "to wast and consume the evil vapours within the chamber, for the breath of man may putrify the air within the chamber"; but he warns us not to go too near to a fire, because it dries up a man's blood. Saint Ignatius, in his constitutions, both for reasons of decency and for the sake of health, forbids his religious to sleep with open windows, and Andrew Borde, in Queen Mary's reign, also thinks it unwise.

### Nursing in Paris Hospitals.

### C.—THE NURSING SISTERS.

### III.—Comparative Merits.

The relative merits of the Sisters as compared with the new laics come under two chief heads, that of character and of cost. The question of comparative cost I will treat in the next article, and will now confine myself to questions of character.

Firstly, I will again allude to the subject referred to in my first article in Series A, that of the popular misapprehension about the functions of the Sisters in Paris hospitals under the old system. They were not the ordinary nurses, but only followed the *rôle* of matrons. The case has never been better put than in the paper on hospitals contributed by the late M. Léon Le Fort to that monumental "Paris Guide" issued for the great Exhibition of 1867, a work written in its various departments by the foremost authors and specialists of France. Dr. Le Fort's article was quoted against him in the heat of the laicisation campaign, but this is only evidence of its acumen and importance. Treating of the nursing Sisters, he says: "The first duty of a writer is to tell the truth, and whatever disfavour may be brought upon us from many of our readers by what we have to say about the duties of the nuns in the hospitals of Paris, it is impossible for us not to explain that their *rôle* is far from that which is attributed to them by prejudice, and which is after all but the souvenir of times long gone by. Their *rôle* does not consist in attending directly to the sick; it is not the Sister who attends the patient but the medical student; and if during the day it is necessary to renew the dressing, to apply poultices, leeches, &c., it is then the male or female nurse who takes the place of the student. The spoonful of medicine which must be given each hour is administered by the nurse. If a dirty sheet has to be changed, a patient to be washed, it is the nurse who does it. The nun is the general supervisor; she partitions the food which is distributed by the nurses; she regulates the reports with the linen-room, and watches over the discipline and order of the ward. The *rôle* of the nun was quite different, if we go back to the statutes of 1536 emanating from ecclesiastical authority: 'Doresnevant pour esviter les occasions de mal, se trouveront et n'y aura aucune personne seculières de quelque sexe ou condition qu'elles soient, au lavoir à aider à faire ou à laver la lexive du linge et aultres quelzconcques mundations de choses, qui soit mesmes à porter des draps, linges, boys ou aultres choses, &c.' To-day at the Hôtel Dieu alone there are 134 male and female laic nurses."

As one director put it to me, the laicisation was, after all, only a change of sergeants and corporals; the rank and file remained the same. This statement must be modified in certain respects, however, for there have been in some places a kind of lower order of Sisters employed, called Sœurs Converses, as at the Paris Children's Seaside Hospital at Berck-sur-Mer, near Boulogne. There the laicisation was particularly expensive, as the premises had been constructed entirely for Sisters, under the auspices of the Empress Eugenie. The country people about that part of Picardy considered laicisation as a fine thing, however, as it gave many of their women folk new jobs at the Government expense. I am afraid the poor children patients, however, do not entirely share this advantage of the change.

Of course, the administrative character of the Sisters is both hotly assailed and as hotly defended. Dr. Bourneville, the great apostle of laicisation, gives as his chief charges against them that they consider themselves as owners of the place wherever installed, with liberty to do as they please, disposing of hospital beds in favour of their particular protegés, and also that they are guilty of cheating the public purse by bearing false entries of patients on the rolls, and that they make many mistakes, sometimes with fatal consequences, owing to their wilful ignorance in the technical part of their duties. I must say that these are very serious charges, and that the doctor has produced very little evidence to show that such evils have been general, although perhaps existing in a very minor degree in some few instances.

As I have remarked, the Sisters have had a very long trial; in fact, almost as many centuries as the laic matrons have had years. The survey of the centuries is certainly a rather noble record for the Sisters. Thus for a hundred years, from 1531 to 1631, I find recorded but three official complaints against the Sisters of the Hôtel Dieu, and these three not all of serious character, the first year being in relation to having too many servants, while in 1569 they were accused of wasting food. The worst charge was in 1559, when poor bed-service was alleged, but probably soon remedied.

I have touched in Series A on the question of the characters of the new laic nurses, and have remarked on some of the faults found, especially in the first years, such as stealing allowances to patients, drunkenness, &c. Of course, no such faults would be long possible with any Sister. The discipline of her order would soon remove her from any chance of repeating any such offence. Another vice with the laics I have personal testimony regarding, which is certainly unknown among the Sisters. An English Protestant clergyman, resident in Paris, informed me that he never sent any charity patients to a French Paris hospital nowadays without giving them small sums of money to tip the nurses with. I fear a little money goes a long way in the Paris hospital ward of to-day. I will further allude to this matter in my article in the series on "Popular Estimation" of the Sisters.

I would by no means wish to be understood as making any universal charge against the new laics, or to deny that there are a great portion of them spotless of any such charges as those above mentioned, but I have to state facts as I find them. As one of the laic advocates has remarked, the lay nurses after all undergo the most dangers, and devote the most time to the repulsive and exacting duties, and certainly a great portion of them must be as much endowed with the true martyr spirit as any Sister can be. Edmund R. Spearman.

### Where to Go.

The New Gallery.—The autumn exhibition at the New Gallery will consist of the works of living French artists, and a collection of pictures and objects lent by Signor Bardini, of Florence. The galleries will be open to the public on Monday, October 3rd.

### Antiseptics for Nurses.

By a Medical Woman.

### XVIII.—SOAPS AND OINTMENTS AS DISINFECTANTS.

Disinfectant soaps have long been in great favour with the general public, which is apt to attach much importance to their use, and it will consequently be useful to consider how far this reliance is justified, and to what extent they should be considered disinfectant. Dr. Rideal has recently published an account of some investigations into the properties of these soaps, and the following is a summary of conclusions come to after very careful and thorough inquiry. Although our knowledge of disinfectants has increased considerably of late years, there has been little or no advance as regards disinfectant soaps, and manufacturers are for the most part contented to keep to those which were introduced many years ago, and which have consequently become well known to the public, and selected when there is a supposed need for a disinfectant soap. The germicidal properties of these soaps have not been scientifically tested as regards definite micro-organisms, and the selection of the particular disinfectant to be used has been left to the manufacturer, regardless of the fact that some disinfectants which have valuable properties as lotions, powders, &c., are quite unfitted to be combined with fats, &c., and manufactured into soaps. Now that the need for thorough disinfection in all cases of infectious diseases is becoming more and more recognised, it is to be hoped that more disinfectant soaps will be made in which the quantity of active disinfectant is definite and constant, and that those of uncertain composition will be ousted. It is therefore important for the soap manufacturer to carefully select his disinfectant, ascertain its purity and efficiency, and add it in the proper proportion to the soap, while the exact amount of such ingredient should be noted on each wrapper. The conditions which obtain when a disinfectant soap is used are very different from those of ordinary disinfecting. The time of contact is much too short, and as this time of contact is so short, it is necessary that the percentage of active ingredients should be high; but when the disinfectant employed is readily soluble in water, actual contact of the infected parts with the disinfectant cannot be attained in the limited time given to washing. In coal tar soaps, and those containing oils which are not very soluble in water, although the disinfectant is modified by the soap, the actual time required for the destruction of the micro-organisms in the affected area is not certain, as organisms vary very greatly in their powers of resistance, and as many of them are very difficult to destroy, and even when a soap contains an approved disinfectant, it must be present in a larger amount than that necessary to destroy the most resisting micro-organisms. The basis of a medicinal soap is important, since the medium itself must have no disturbing action, and hence care must be exercised to secure what is known as chemical neutrality, or the due combination of the fatty acids and alkalis that go to make soap, so that there is no excess of either alkali or fatty acid, which are said to produce irritation and inflammation respectively, and raw materials must be carefully selected as neutral, uncoloured, and almost inodorous glycerides. The alkali of commercial soap is soda, but potash or soft soap is also used, and is generally made with linseed oil, and has a pale brownish-green colour, and is considered to be especially beneficial in some skin diseases. The chief objection to commercial soft soaps is that they are not freed from uncombined alkali and glycerine by the process of "salting out" or adding a given quantity of common salt or strong brine, in which the soap is insoluble and its constituents separate, the soap collecting on the surface, and the spent lye settling to the bottom; hence when salting out is not done the soap retains a large quantity of water, all the glycerine of the original fat, and the saline impurities of whatever alkali has been used in its manufacture, some of which may interfere with the action of the disinfectants used. To avoid these difficulties a specially pure soft soap, called savonal, has been prepared, made by saponifying olive oil in the cold with alcoholic potash, and sufficient fatty acids to neutralise the resulting solution are added carefully, and finally the alcohol is distilled off, and the result is savonal. It has been found that super-fatted soaps or those which contain an excess of the fatty acids are not so suitable for medicated soaps as those having a slight excess of alkali, and that the presence of free oil or fat interferes with antiseptic action and impede the germicidal action of such disinfectants as mercuric chloride, phenol, &c. Moreover, Koch has shown that carbolic acid dissolved in olive oil, forming what is known as carbolised oil, has no antiseptic properties, and that some micro organisms live in oil longer than in aqueous solutions, and hence all oils and fats used in ointments and soaps must be sterilised by heat, which is usually done in course of manufacture; and consequently soaps themselves have considerable antiseptic power, and there is no doubt that prolonged contact with soap renders surfaces practically sterile, though under ordinary circumstances common soaps fail to do so.

Sulphur blends well with soaps, though even in the form of milk of sulphur its action is very slow owing to its insolubility, but it has been proved useful in skin diseases. Boracic acid is of no use in soaps, and metallic salts also are of very little use, as they can only be introduced into soap in very small quantities, and are precipitated in an insoluble form. Carbolic and cresylic soaps are said to be 10 per cent. strengths, but they are not uniformly so, and all forms of these soaps have a very strong odour, which is often a disadvantage. Several varieties are advertised as of "delicate odour," and "not unpleasant in any boudoir," but although the cresols have a higher disinfectant action than phenol, they still have a distinctive odour, if in effective proportions, so that a soap of the tar order, however much disguised with eucalyptus, &c., cannot be free from more or less of a tarry odour. A number of toilet soaps are advertised in conjunction with the names of various disinfectants, but contain such an infinitesimal amount of them that they are quite useless as germicides, and their use is even dangerous as giving a sense of security which does not exist. The essential oils are of little use as disinfectants in soaps, as they must be used in large quantities to be really efficient, but they are useful in very minute quantities when a scented soap is desired. Such volatile disinfectants as phenol, camphor, thymol, &c., undergo considerable loss when in or added during re-melting in the ordinary way, and consequently the quantity present is uncertain, and hence they should be introduced in a special way. In experiments made with clove-oil and carbolic soap, it was found that oil of cloves, when present in a soap, has very little antiseptic action, and that both it and carbolic soap have only an antiseptic action which is equal to, but does not exceed, that of ordinary curd soaps. "Salufer" is the name given to a patent soap in which the disinfectants are fluorides and silico-fluorides, the antiseptic properties of which were discovered many years ago, and the whole class was patented under the name of "Salufer." Fluorine itself is said to be more active than chlorine, but it is not likely to be made available, as its action is very intense, and its preparation difficult. Sodium silico-fluoride is the form that is used, and is prepared as a powder, which is sparingly soluble in water, but is said to be irritating to wounds, and non-toxic. It is prepared in cubes of definite weight, with which a lotion of known strength can be prepared by simply dissolving in water.

### Plague Nursing.

By a Nursing Sister.

I beg to send you a short account of the plague work in Bombay during the last epidemic. I was not working there in the earliest part of it, as when I first came out to India I was at a place called Sholapore, in the Deccan, where the epidemic only lasted a short time—about four months at most. About the beginning of February I was transferred to Bombay, where the epidemic was fast reaching a height.

At the time I began working in Bombay there were, I think, about seven plague hospitals open nursed by English sisters. I was put to work in the Arthur Road Hospital, which had originally been the small-pox hospital, and is now converted into the permanent plague and kelapsing, or famine fever hospital. Here the patients were absolutely pouring in, and I may say almost as fast pouring out—the mortality was so high. I really believe myself that numbers of them died of sheer terror. I had the male acute ward for a time, which held about 43 patients. Of course we had not so much to contend with as we had had in our previous experience, where there was not room for the patients, &c.; here things were in very fair working order, as the English sisters had been there for nearly a year. The wards had wooden boarded walls, with *packa* (real) windows, and tiled roofs. The floors, with one exception, were mud—the exception was the female ward, in which we had the luxury of a stone floor, which could be washed all over every day.

The patients had very comfortable beds, made on the principle of cane chairs, two nice white cotton blankets to act as sheets, a pillow, and a good warm woollen blanket over them. Into the acute ward the patients were brought when they first came in. If they lived three or four days they were then transferred to what was called the convalescent ward; and about the ninth or tenth day, if they went on fairly well and seemed likely to recover, they were transferred again to the convalescent hospital at the Government House Park, about two miles further out of Bombay than the hospital itself.

We used to go on duty at 7 a.m. one day remaining until 12, then going off duty and returning at 4 until 8 p.m., when relieved by the night nurses. The next day we were on duty from 7 a.m. until 4 p.m., when we went off for the rest of the day. Later on, as the work got lighter, we went on duty from 7 a.m. until 1.30 p.m. one day, and the next from 1.30 till 8 p.m.; the night sisters being on from 8 p.m. until 7 a.m. next morning. The night duty has remained the same ever since we came out, and our turns come round about every third week. I think we are all agreed that the night duty hours are much too long. At the time the epidemic was at its worst it was simply a case of remaining eleven hours in a ward with 40 to 45 patients all raving and shrieking like maniacs the whole time. Of course, to nurses at home the duty hours do not sound long; but the climate, and the fact that we have to be out in the greatest heat of the day when no other Englishwoman goes out of doors at all, must be taken into great consideration.

It seems to me that though things are very much improved now, looking back on the past year we had all sorts of odds to work against the whole time. First, when we came out we were landed in hospitals to nurse people and train ayahs and ward boys whose language we did not understand half a dozen words of. Next we had all sorts of absurd superstitions to battle against; one of their ideas is that as a punishment for the tarring of the Queen's statue, Her Majesty requires the livers of so many natives to avenge the insult, therefore the reason of the plague coming upon them; and they also think that we do not wish them to recover, but rather assist in helping to get rid of them. One day a woman was brought into my ward suffering from a very bad form of plague, from which she eventually died. Immediately she was laid upon the bed preparatory to being washed, she jumped up, and with an awful shriek declared that nothing would induce her to lie upon that bed, as she knew she should die if she did, because the bed was poisoned. Another time, whilst in one of the observation camps, I heard one man tell another most impressively, that however ill he might be he was not to go to a certain plague hospital, "because," he said, "they give you red medicine there that kills you." Another grave difficulty lies in the fact that plague patients invariably refuse all nourishment or medicine, and it is a case of first persuading, which at one time was absolutely useless, and then forcing, which seems to me more than cruel, as the poor things struggle so that afterwards they are quite exhausted. Of course, in many cases we have to resort to feeding them with nasal tube and very often indeed with nutrient enemata.

A great feature of plague is the violent delirium and fear which seizes the people, and nobody can have the faintest idea of the awful noise there used to be in the wards when the epidemic was bad. Another much worse evil than the noise was that in many cases it was quite impossible to keep the patients in bed, and so we had to resort to the means of tying them down, or else they would have run away. Of course, it is a thing that every true nurse hates to do, but it was a case of absolute necessity.

The worst forms of plague are (1) the pneumonic plague, from which the patient never recovers—this must not be confused with plague with pneumonia, which is a very common thing. Pneumonic plague is far and away the most infectious form of the disease; there is very seldom an external bubo, but all the symptoms of very bad pneumonia, very quick respirations. I have known a case where the respirations went up to 120, and the pulse was less than 60—cough and expectorating blood. (2) This form is that which has no external bubo, often low temperature and quick small pulse, very often suffering from most acute internal pain; from this I have known a patient seem fairly well one minute, raise himself ever so slightly and fall back dead the next. (3) Also another most frequently fatal form is that with buboes or glandular swellings in the neck. These swellings are hard and tense, and no fluctuation is obtained; on incision no pus as a rule is found; these cases die in a very short time of suffocation. I have only enumerated the worst forms according to my own experience. Of course very often the patient has several of these buboes. I myself remember a case of a little girl of nine years old who had no less than eleven buboes, all of which suppurated. She recovered perfectly. Empyema is very common, and I have seen several very bad pelvic abscesses. Of course, as about everything else, there are many varieties of opinion as to the treatment of these buboes. Some doctors order poultices or hot fomentations; others ice bags to the bubo until it softens. My experience is that they often do a great deal better if they are left absolutely alone until they are ready for incision. We get some most marvellous cases of high temperature. I had a patient brought in the other day with temperature of 105·8. He was at once put into an ice-pack, with an ice bag to the head and another to the back of his neck. Even while in the pack the temperature rose to 108, and gradually, keeping him in the ice-pack for forty-eight hours, we got the temperature down to 101, but a few hours afterwards he collapsed and died.

After March was over the epidemic began rapidly to decline, and until the latter part of July we had very few cases. Then they began to come in pretty frequently, and now you will see that the epidemic is increasing rapidly. So we are all looking forward again to six months of real hard work.

### The Book World for Women and Nurses.

[We invite Correspondence, Criticism, Enquiries, and Notes on Books likely to interest Women and Nurses. Address, Editor, THE HOSPITAL (Nurses' Book World), 28 & 29, Southampton Street, Strand, London, W.C.]

THE CARE OF CONSUMPTIVES. By W. H. DAW, M.R.C.S., L.R.C.P. Pp. 79. (London: The Scientific Press. 1898. Price 1s.)

This little book is designed for the use of nurses, and, we have little doubt, will fulfil its purpose admirably. It is not, as so many books for nurses are, burdened with unnecessary pathological detail, but contains in brief compass most of that which a nurse in charge of phthisical cases will find it advantageous to know. At the same time, there is sufficient explanatory matter to give an intelligent nurse interest in the details of the treatment she may have to carry out. The directions given for action in case of emergencies, such as hæmoptysis, are concise and clear, and sufficiently bespeak the author's familiarity with his subject. One or two little nursing hints, such as the use of an abdominal pad in diarrhœa, and eau de cologne or aromatic vinegar for sponging, might with advantage have been inserted; but, on the whole, little has been omitted. The chapter on the nurse's care of herself is valuable, and that on "Prophylaxis" will serve to enlist nurses as missioners for the "New Crusade." Every nurse who has not had the advantage of experience at a special hospital should, when called to a case of phthisis, slip this little book in her bag, for, common disease as phthisis is, there is much in its nursing not taught in the wards of a general hospital.

TOURIST GUIDE TO THE CONTINENT. Published at 30, Flee, Street, E.C.

This excellent little guide to the Continent will, we venture to predict, prove of much service to the summer tourist. For the modest sum of sixpence a great deal of valuable information is here provided. Among the most noticeable features contained in the book are particulars of the new express service to Norway, Denmark, and Sweden, viâ the Royal Mail Harwich Hook of Holland route; a series of continental maps, a chapter upon cycling routes in Holland, Belgium, and Germany, and a chapter, "Dull Useful Information," giving particulars as to the cost of Continental travel. We congratulate the editor, Mr. Percy Lindley, both as to the excellent manner in which he has arranged his matter, as also to the illustrations and maps.

CHAVASSE'S ADVICE TO A WIFE ON THE MANAGEMENT OF HER OWN HEALTH. 14th Edition Edited by FANCOURT BARNES, M.D. 8mo., 306 pp. (London: J. and A. Churchill. 1898. Price 2s. 6d.)

The fact that this book has reached the 290th thousand is sufficient evidence of its continued popularity among those for whom it was written. It evidently holds its own among younger rivals; and the reason for this is not far to seek, for the book is very clearly written, and the directions given are, for the most part, sound and reliable. The great number of poetical quotations which are found will perhaps prove irksome to some readers; but to others they may form a welcome break in the more serious matter. The most objectionable feature in the book is the enormous amount of repetition. The editor of this edition recognises this, but has left it untouched, as being, in his judgment, a fault on the right side. We venture, however, to disagree with him, and to suggest that if he has the opportunity of revising another edition, he will render the book more attractive and no less useful by pruning some of the redundancies. The colloquial style of the author occasionally verges on the ridiculous; for instance, after advising the wearing of a flannel vest, both winter and summer, he proceeds, "scarlet is, in such a case, a favourite colour, and may be selected for the purpose." Again, speaking of flatulence, "The wind rumbles about the bowels outrageously; first in one place and then in another, and then rising in volumes to her throat almost chokes her." Part of the chapter on menstruation does not give the impression of having been written by a medical man. For instance, "Too scanty menstruation is a frequent cause of sterility." "Deficient menstruation is a frequent cause of the 'whites.'" It is new also to learn that at quickening "there is a sudden increase in the size of the abdomen." There is no reason why a "popular" book should be inaccurate, and seeing how good most of this book is, we shall hope to see the next edition better revised.

CHAVASSE'S ADVICE TO A MOTHER ON THE MANAGEMENT OF HER CHILDREN. 15th Edition. Revised by GEORGE CARPENTER, M.D. 8vo., 430 pp. (London: J. and A. Churchill. 1898. Price 2s. 6d.)

This work, like its companion, "Advice to a Wife," has attained to a green old age, this edition being the 240th thousand. It is, therefore, scarcely necessary at this time of day to enter into a detailed description of it. The arrangement of the book is in the form of question and answer, an arrangement which is conducive to a clear didactic style. We must congratulate Dr. Carpenter on the way he has done his work; the book is well brought up to date, as is particularly shown in the passages on the diet of infants. We feel sure that if all mothers were to read the book in the spirit in which the author desired it, "thoughtfully and carefully studied until its contents in all their bearings be completely mastered and understood," their children would have occasion to "rise up and call them blessed." More than this, the care of infants and young children is a subject which most medical students learn little about; and young practitioners often find, to their chagrin, that they are comparatively "at sea" in a department of practice which is most important both to themselves and their patients. To students and practitioners we would venture to commend this work; they as well as the mothers for whom it was written will find a storehouse of valuable suggestions and sound teaching, which are none the less useful for being cast in a homely and simple style. We may call attention to a feature of the book which is non-medical, but in every way admirable, namely, the part of it which refers, generally incidentally, to the moral aspect of the care and training of children. We heartily wish for the work a continuance of the popularity which it has hitherto enjoyed.

### Novelties for Nurses.

### AN EXCELLENT TEAPOT.

NUMBERLESS contrivances to prevent the unnecessarily long infusion of tea in the teapot have already been introduced, but so far none have been free from drawback. In the Bellawattee Patent Teapot difficulties have been in our estimation most satisfactorily overcome. The plan adopted is simple, but effectual; a perforated receiver for the tea leaves is suspended by a chain through the knob which surmounts the cover of the teapot. Whilst the tea is infusing it rests in the perforated receiver in the water; when desired, the receiver is drawn well up out of the water by the chain, and remains fixed and suspended within the cover of the teapot. The necessity of taking the cover off, removing the receiver, and selecting some suitable place to deposit it, which is most inconvenient, is thus avoided. Most nurses are lovers of tea, and this teapot will, therefore, be much appreciated by them. The showrooms of the Bella Wattee Company are at 244, Oxford Street, where all sorts of pretty teapots may be seen. Such a teapot would form an excellent present.

### Everybody's Opinion.

[Correspondence on all subjects is invited, but we cannot in any way be responsible for the opinions expressed by our correspondents. No communication can be entertained if the name and address of the correspondent is not given, as a guarantee of good faith but not necessarily for publication, or unless one side of the paper only is written on.]

### THE HOUSEMAID OR LADY NURSE.

"X Y Z" writes: Since the correspondence on "Ladies v. Gentlewomen" was started the subject seems somewhat to have changed, and the question now appears to be, "From what class of women is it most desirable to recruit our hospital nurses?" It is difficult to discuss the subject without appearing arrogant and uncharitable, but there is one side of it which is generally ignored—the social side, one might call it. "Servant class nurse" is not a happy term, but one must use it for want of a better. There are great objections to the employment of this class of women in a large general hospital. One is the extreme difficulty of preserving a proper tone and discipline in a ward, especially in a men's ward, where the nurses are more or less of the same class as the patients. I am writing from several years' experience, both as nurse and sister. During my training I was sent to many wards in succession to take "nights off," and the behaviour of the patients told me at once whether their nurse was a lady or a housemaid. The night nurse being the only one in the ward for between ten and twelve hours, has great influence; the patients, being naturally less under constraint in the sister's absence, will take their tone from the night nurse. I was once helping one of the "servant class" with the beds, when one of the patients whose bed we were doing made a low joke, at which, to my disgust, this woman giggled. No doubt, in her rank of life that sort of thing passed for humour, and she saw nothing wrong. Had she been a lady she would have reproved the patient and gained the respect of the whole ward. As it was, she had lowered its tone and the men's opinion of the nurses generally. The same difficulty occurs with regard to the relations between the nurses and the hospital servants. These latter are usually anything but respectful, and when a nurse walks arm-in-arm with a housemaid or chats in a confidential manner with a porter, as I have frequently seen the "servant class nurse" do, it does not improve matters, and makes the position of those of her fellow-nurses who do not relish the society of the maids and porters very unpleasant. These same nurses will "walk out" with the students when off duty. I know they think no harm of these things, as they are the every-day custom of their class; but, all the same, they do undoubtedly lower the nursing profession in the eyes of many. Then, as to the intellectual side of their work, these nurses, as a rule, dislike lectures and hardly ever read. I know it is the fashion rather to sneer at a nurse who is intellectually as well as mechanically trained, and to think that a thorough knowledge of the theory of her work is a distinct bar to a nurse's chances in its practice. It is supposed that a nurse who "knows all about knee jerks" is therefore incapable of "making a bed" (which, by the way, appears to the lay mind to represent the sum total of a nurse's duties, with the addition, perhaps, of occasionally "smoothing a pillow"). Still, the fact remains that hospital nurses nowadays are expected to know much more than the mechanical side of their work. They must be able to read Latin prescriptions and directions, to be acquainted with the action of the different drugs and the symptoms of an overdose; to be able accurately to calculate and administer fractions of a grain from solutions of different strengths. They must be familiar with and able to recognise the importance of the symptoms of various diseases. In some hospitals they are required to test for and estimate the amount of albumen, sugar, urea, &c., in urine, &c. It is absurd to expect a domestic servant to be capable of this sort of work. Good character, sympathy, and gentleness are essential in a nurse, but so are the power of governing a ward, the intellect to "read, mark and learn," and the good breeding which, while courteous to all, is yet familiar with none.

*** There can be no doubt that the points raised by our correspondents are very important. But the question is fraught with difficulties. There is an increasing demand for well-trained women of the housemaid class to act as nurses in private families owing to the belief that their requirements will be simpler and that they are less likely to disarrange the household. If this class are excluded from hospital training, the demand cannot be supplied, and where the objection to employing the lady nurse is very strong an opening for the untrained Gamp will once more be made. We fully agree with our correspondent that ladies of superior education should be selected for posts of administration in institutions, and this should be understood. At Guy's Hospital the rule of promoting only lady probationers to be sisters of wards was a wise one on this account, and had the training demanded been longer it would have been a good arrangement. We feel sure that the tone of a ward will not suffer if the sister in charge is vigilant, although all the nurses be not drawn from the more educated classes.—Ed. *T. H.*

### Appointments.

Davos Invalids' Home, Switzerland.—Miss Gertrude Washbourn has just been appointed Lady Superintendent to this institution. Miss Washbourn was trained at the Guest Hospital, Dudley, and at the Seamen's Hospital, Greenwich. She then acted as night superintendent at the Kensington Infirmary, and more recently as assistant matron at Warneford Hospital, Leamington.

The London Temperance Hospital.—Miss Amy Lucas has been appointed Matron of this hospital. Miss Lucas was trained at the General Infirmary, Leeds. She acted as sister at the Radcliffe Infirmary, Oxford, as assistant matron of the Gore Farm Hospital, and from 1894 until the present time has been sister-housekeeper at the Temperance Hospital.

Convalescent Home, Wotton-under-Edge.—Miss M. I. Tayler, who has been appointed Matron to this institution, was trained and was for eleven years sister at the Bristol General Hospital, not at the Bristol Infirmary, as stated in error in our last week's issue.

### Minor Appointments.

The City Hospital, Liverpool.—Miss Valetta Short has been appointed Charge Nurse at the above hospital. She was trained at the Alexandra Hospital for Children, London, and at Addenbrooke's Hospital, Cambridge. She acted as private nurse for the Kent and Canterbury Nurses' Institute, and lastly as staff nurse at the Sanatorium, Hull.

Aberdeen East Parochial Hospital.—Miss M. Lyall has been appointed Superintendent Nurse of the above institution. She was trained and afterwards acted as charge nurse at St. Luke's Hospital, Halifax. During the last eighteen months she has worked as private nurse for the Edinburgh Trained Nurses' Association.

Keighley and Bingley Joint Fever Hospital.—Miss Shepherd has been appointed Sister at the above institution. She was trained at the London Hospital, and afterwards was charge nurse at the temporary hospital at Worthing, and latterly has been occupied in private nursing at Bradford.

Workhouse Infirmary, Bootle.—Miss May Isa Boyd has been appointed Nurse at this institution. She was trained for two years at the General Infirmary, Worcester, having been placed there as probationer at the expense of the Meath Workhouse Attendants' Association.

### For Reading to the Sick.

OUR WORKS IN GOD'S HAND.

**Verses.**

OH, to be nothing, nothing !
Only to lie at His feet,
A broken and emptied vessel,
For the Master's use made meet.
Emptied, that He may fill me,
As forth to His service I go;
Broken, that so unhindered
His life through me might flow.

Oh, to be nothing, nothing !
Only as led by His hand—
A messenger at His gateway,
Only waiting for His command.
Only an instrument ready
His praises to sound at His will;
Willing, should he not require me,
In silence to wait on Him still.

—*G. M. T.*

Thus, dishonouring not her station,
Would my life present to Thee,
Gracious God, the pure oblation
Of divine tranquility. —*Wordsworth.*

The deeds we do, the words we say—
Into still air they seem to fleet,
We count them ever past;
But they shall last!
In the dread Judgment they
And we shall meet. —*Keble.*

**Reading.**

Suppose an angel were sent down to tell us this morning that he was commissioned to take all our work under his charge to-day, that we might just be easy about it, because he would undertake it, and his excellent strength and wisdom would make it all prosper a great deal more than ours, how extremely foolish it would be not to avail ourselves of such superhuman help. What a holiday it would seem, if we accepted the offer, as we went about our business with the angel beside us! What a day of privilege and progress! And how we should thank God for the extraordinary relief His kindness had sent! Far higher is our privilege this day; not merely permitted, but pressed upon us by Royal Commandment. "Commit thy works unto Jehovah." Yet this is but the third strand of a golden cord which is strong enough (if yielded to) to draw us up out of all the miry clay of "the pit of noise," where the voices of fear and anxiety and distrust make such a weary din. We are to commit the keeping of our souls to Him; then we shall be ready for the command to commit our way unto Him, and then our works. Then, having obeyed, we may exchange the less confident expression "Unto God would I commit my cause," for the bright assurance, "I am persuaded that He is able to keep that which I have committed unto Him."—*F. R. H.*

### Presentations.

MISS A. J. LAST, superintendent nurse at the Stapleton Infirmary, Bristol, was the recipient on the 23rd inst. of a handsome Royal Worcester china and gold biscuit jar, presented by the medical officer and nurses on her leaving that institution to take charge of the new infirmary shortly to be opened by the Wakefield Guardians. Many kind wishes were expressed that she would meet with every success in her future sphere.

### Notes and Queries.

**The contents of the Editor's Letter-box have now reached such unwieldy proportions that it has become necessary to establish a hard and fast rule regarding Answers to Correspondents. In future, all questions requiring replies will continue to be answered in this column without any fee. If an answer is required by letter, a fee of half-a-crown must be enclosed with the note containing the enquiry. We are always pleased to help our numerous correspondents to the fullest extent, and we can trust them to sympathise in the overwhelming amount of writing which makes the new rules a necessity.**

**Every communication must be accompanied by the writer's name and address, otherwise it will receive no attention.**

*Miss Kate Marsden.*

(1) I should be glad if you could give me some information concerning Miss Kate Marsden, whose whereabouts I cannot trace since March, 1893. She was spoken of as "The Leper Lady."—*M. Smith.*

We know nothing of the lady in question at the present time. Why not apply to the Charity Organisation Society, 15, Buckingham Street, Adelphi, W.C.

*Winter Abroad.*

(2) Can you inform me the best way of obtaining a patient to winter abroad? I am fully trained.—*F. G. Smith.*

Your best plan would be to advertise for a position such as you require in the medical and daily papers.

*Hospitals Training Probationers.*

(3) Are there any hospitals in England where they receive probationers and give them salary during their training?—*G. G.*

Yes, many. Get a copy of "How to Become a Nurse," by Honnor Morten (London: Scientific Press), where you will find a list of hospitals training probationers, together with all particulars.

*District Maternity Nursing.*

(4) Which is the cheapest and best way of training for district maternity nursing where certificate is given?—*H.*

Write to the secretary of the London Obstetrical Society, 54, Berners Street, London, W., for full particulars of their examinations. The diploma of this society, which is granted to candidates successfully passing their examinations, should be held by all midwifery nurses.

*Address Wanted.*

(5) Can you tell me the address of the Alpine Wool Company? Also which days the Royal College of Surgeons is open to visitors?—*F. H.*

We are sorry not to be able to ascertain the address required. Perhaps some of our readers may be able to help. The Royal College of Surgeons Library is open each week day except Saturday from eleven a.m. to seven p.m., during the months from January to July and October to December. On Saturdays it is closed at one o'clock. Members have the privilege of introducing a visitor.

*Materia Medica.*

(6) What is the price of a Materia Medica, and what would be the best book for me to have so as to be able to judge the amount of a drug I ought to give to children, for instance?—*A. F. R.*

We give you the names of three books, either of which would give you the information you require: "Elements of Materia Medica and Pharmacy," by A. W. Gerrard, F.C.S., price 8s. 6d.; "The Official Materia Medica," second edition, by F. T. Roberts, M.D., F.R.C.P., price 7s. 6d.; "Pharmacy and Dispensing," by C. J. S. Thompson, price 1s. All these may be obtained through the Scientific Press, 28 and 29, Southampton Street, Strand, London, W.C.

*Open-Air Treatment of Consumption.*

(7) Will you kindly let me know the best book to get for information about the open-air treatment of consumption? *Florentine.*

Much recent information about the sanatorium and open-air treatment of consumption is to be found in the Special Tuberculosis Number of the *Practitioner*, which was issued in June of this year. In the next issue of THE HOSPITAL, which will be our Special Autumn Number, there will be a special article on Winter Health Resorts and other articles in which the treatment in question will be dealt with.—ED. T. H.

*Queen's Nurse.*

(8) I am anxious to have information of the Queen's Jubilee Fund for training women to nurse the sick poor in their own homes.—*E. M. P.*

Write to the Secretary of the Queen Victoria Jubilee Nurses' Institute, St. Catherine's Hospital, Regent's Park, N.W., who will give you all particulars.

*Seaman's Hospital.*

(9) Kindly tell me if I can get into either a naval or seamen's hospital without a premium, and where to apply? Have had one year's general training and four private nursing.—*M. K.*

The Seamen's Hospital, Greenwich, receives probationers without premium. When applying, state what previous training you have had, and the matron will then be able to give you all particulars.

**ANSWERS REQUESTED.**

*Dandruff.*

(10) In reply to a query of a correspondent in "The Mirror" of August 13th, "L. B." kindly writes us that an anti-scurf pomade is made by Mr. Walker, hairdresser, 119, Newington Green Road, London, N. "L. B." writes that she herself has tried the pomade and believes it to be really efficacious

*Nurses' Hospital Lectures Open to Ladies.*

(11) A correspondent inquires whether there is any hospital in London or the suburbs where ladies are allowed to attend the lectures or classes given to the nurses. Perhaps one of our readers may be able to supply this information.

## Figures and Tables

**Fig. 1. Fig. 2. Fig. 3. Fig. 4. Fig. 5. Fig. 6. Fig. 7. f1:**
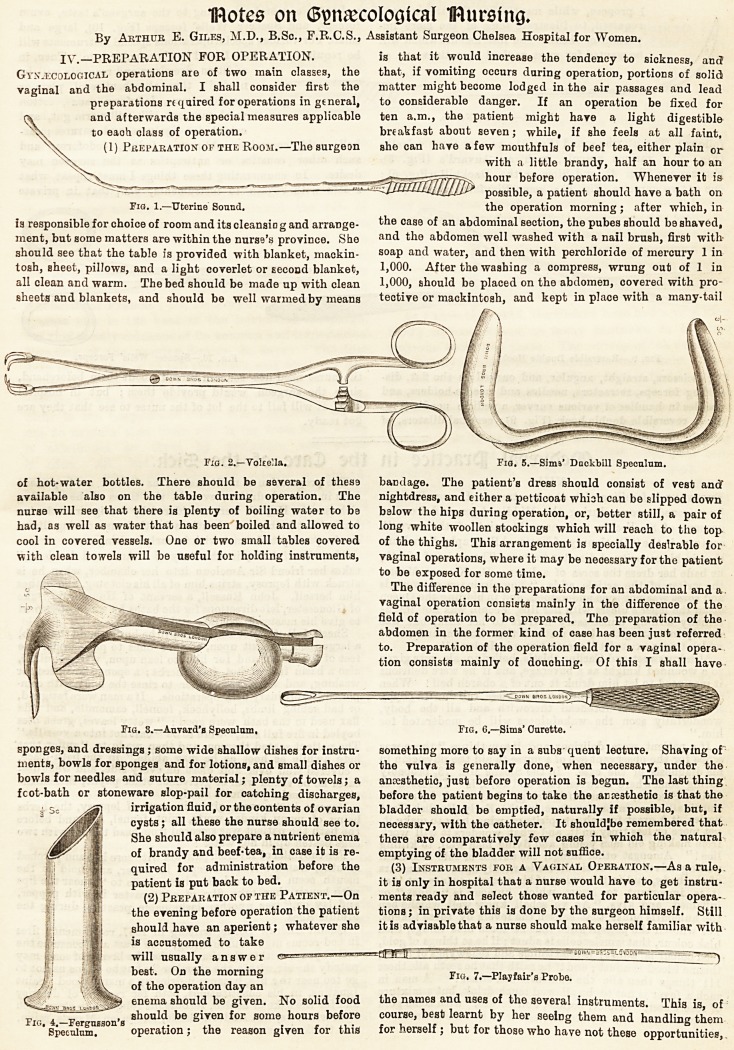


**Fig. 8. Fig. 9. Fig. 10. f2:**